# Modification of Immobilized Titanium Dioxide Nanostructures by Argon Plasma for Photocatalytic Removal of Organic Dyes

**DOI:** 10.3390/molecules24030383

**Published:** 2019-01-22

**Authors:** Hamid Reza Khaledian, Pezhman Zolfaghari, Vahide Elhami, Mostafa Aghbolaghy, Sirous Khorram, Afzal Karimi, Alireza Khataee

**Affiliations:** 1 Department of Chemical Engineering, Faculty of Chemical and Petroleum Engineering, University of Tabriz, 51666-16471 Tabriz, Iran; khaledian.hamidreza@yahoo.com (H.R.K.); pegmanvista@gmail.com (P.Z.); v.elhami68@gmail.com (V.E.); 2 Department of Chemical and Biological Engineering, University of Saskatchewan, 57 Campus Drive, Saskatoon, SK S7N 5A9, Canada; aghb.s@usask.ca; 3 Research Institute for Applied Physics and Astronomy, University of Tabriz, 51666-16471 Tabriz, Iran; skhorram@tabrizu.ac.ir; 4 Faculty of Advanced Technologies in Medicine, Iran University of Medical Sciences, 14496-14535 Tehran, Iran; 5 Research Laboratory of Advanced Water and Wastewater Treatment Processes, Department of Applied Chemistry, Faculty of Chemistry, University of Tabriz, 51666-16471 Tabriz, Iran; 6 Health Promotion Research Center, Iran University of Medical Sciences, 14496-14535 Tehran, Iran

**Keywords:** cold plasma, TiO_2_, surface modification, nano-catalyst, photocatalysis

## Abstract

The aim of this study was to modify surface properties of immobilized rutile TiO_2_ using Argon cold plasma treatment and to evaluate the performance of the catalyst in photocatalytic elimination of synthetic dyes in UV/TiO_2_/H_2_O_2_ process. The surface-modified TiO_2_ was characterized by XRD, EDX, SEM, UV-DRS and XPS analyses. Response surface methodology was adopted to achieve high catalyst efficiency by evaluating the effect of two main independent cold plasma treatment parameters (exposure time and pressure) on surface modification of the catalyst. The increase of the plasma operation pressure led to higher decolorization percentage, while the increase of plasma exposure time decreased the decolorization efficiency. RSM methodology predicted optimum plasma treatment conditions to be 0.78 Torr and 21 min of exposure time, which resulted in decolorization of 10 mg/L solution of the malachite green solution by 94.94% in 30 min. The plasma treatment decreased the oxygen to titanium ratio and caused oxygen vacancy on the surface of the catalyst, resulting in the superior performance of the plasma-treated catalyst. Pseudo first-order kinetic rate constant for the plasma-treated catalyst was 4.28 and 2.03 times higher than the rate constant for the non-treated photocatalyst in decolorization of aqueous solutions of malachite green and crystal violet, respectively.

## 1. Introduction

About 1–20% of total annually produced synthetic dyes are released into the effluents of the textile, paper, pharmaceutical, food, and plastic industries [1,2]. This causes considerable environmental pollution via production of hazardous byproducts through hydrolysis, oxidation and other chemical reactions [1,3,4]. Synthetic dyes such as malachite green oxalate (MG) and crystal violet (CV) are mostly non-biodegradable. They are toxic compounds, which due to their carcinogenic, mutagenic and allergenic properties, disturb water ecosystem and affect the immune system of humans and animals [5]. Various biological, physical and chemical methods are used for decolorization of wastewaters [6]. Incomplete degradation of dyes and long residence time are the main drawbacks of physicochemical and biological methods, respectively [7]. Advanced oxidation processes (AOPs) have received considerable attention for complete mineralization of dyes based on generation of hydroxyl radicals that oxidize organic pollutants [8,9]. AOPs such as ozonation, UV/O3, photocatalysis, Fenton, bio-Fenton [10,11], photo-Fenton, electrochemical oxidation [12,13], UV/O_3_/H_2_O_2_, UV/H_2_O_2_ and UV/TiO_2_ have been explored to treat wastewaters [14,15].

Photocatalytic elimination of synthetic dyes using semiconductors such as TiO_2_, ZnO and ZnS has been widely reported in literature [16,17,18,19]. Nina et al. [19] prepared ZnO films for decolorization of 5 mg/L solution of malachite green dye under UV illumination and achieved 96% of degradation efficiency after 210 min. In another study, Fe^3+^-doped TiO_2_ was prepared and its performance was investigated for degradation of 5 mg/L MG solution. With an optimized catalyst preparation process, MG elimination efficiency of 80% was obtained after 100 min of UV illumination [17]. Sadia et al. [20] prepared flower-like ZnO structures to degrade a crystal violet solution with a concentration of 10 mg/L. They reported that under UV illumination by a xenon lamp (300 W), 96% of crystal violet was eliminated within the 80 min of operation. Chiing et al. [21] inspected homogenous decolorization of crystal violet solution by TiO_2_ under a 15 W UV irradiation source and 99.5% decolorization was achieved after 16 h. 

Among many semiconductors, TiO_2_ has been found to be the most effective catalyst for photocatalytic processes due to fast electron transfer to molecular oxygen, non-toxicity, availability, physicochemical stability, and low cost [22,23,24,25,26,27]. Photocatalytic efficiency of TiO_2_ is often enhanced by integration with other nano-structured materials, impurity doping and surface modification/complication sensitizations [28,29,30]. Three recognized crystalline phases of TiO_2_ recognized are anatase, rutile and brookite [31]. Among the three phases of TiO_2_, the anatase phase was proved to be the best choice for photocatalytic processes in the presence of the UV light [32]. However, its larger band gap limited its application in visible light. On the other hand, rutile phase, with a narrower band gap of around 3 eV, has rarely been investigated by researchers due to its lower photocatalytic performance [33,34]. 

As the fourth state of matter, plasma is composed of ions, electrons, radicals, atoms and molecules and has been categorized as low and high temperature plasma [35]. Low temperature plasma is classified as thermal (i.e., quasi-equilibrium) and cold (i.e., non-equilibrium) plasma [36,37]. Cold plasma is advantageous because of low energy consumption, low operational pressure, and clean reaction. This technique of plasma modifies the surface properties of materials, without altering the bulk properties [38,39]. Previously, Jung and et al. deposited titanium dioxide on glass via sol-gel processing and studied the oxygen plasma, which was ignited by radio- frequency (RF) and microwave (MW) under both vacuum and atmosphere conditions. They tested the photocatalytic activity of the catalyst in toluene and phenol removal and reported that phenol was quickly decomposed with RF plasma treatment [40]. An et al. utilized cold hydrogen plasma to modify the properties of anatase TiO_2_ and reported that after 120 min of plasma exposure time, specific surface area increased, and oxygen vacancies formed. As a result, they managed to increase decolorization efficiency of various dyes [41].

In this study, rutile phase TiO_2_ nanoparticles were immobilized on glass plates and their activity for decomposition of malachite green and crystal violet dyes was examined using UV/TiO_2_/H_2_O_2_ process. Table 1 presents the chemical and physicochemical properties of these synthetic dyestuffs. Cold argon glow discharge plasma was applied to modify surface properties of rutile phase nano TiO_2_, which is superior to anatase phase in terms of visible light activity and the potential to be built inexpensively in large quantities [42,43]. Furthermore, Response Surface Methodology (RSM) was employed to explore the interaction between plasma operational parameters and optimize operating conditions to achieve the maximum photocatalytic performance. RSM is an efficient and widely used statistical-based method for multivariate analysis which has been employed to investigate the interaction between operational parameters and reduce the experimental workload in wastewater treatment [44,45].

## 2. Results and Discussion 

### 2.1. Characterizing of the Catalysts

**Figure 1:**
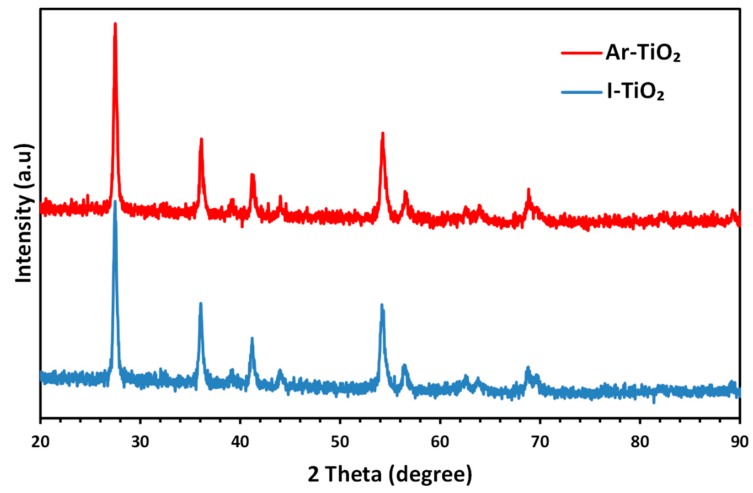
X-Ray diffraction patterns of Ar-TiO_2_ and I-TiO_2_ samples.

Figure 1 presents X-ray diffraction patterns of Ar-TiO_2_ and I-TiO_2_ samples. The characteristic peaks of TiO_2_ appeared at 2θ values of 27.50°, 36.08°, 39.21°, 41.22°, 44.07°, 54.22°, 56.48°, 62.63°, 63.83°, 68.80°, 69.72°, 82.18° and 89.28°, and 27.51°, 36.15°, 39.21°, 41.29°, 44.04°, 54.24°, 56.60°, 62.58°, 64°, 68.86° and 89.40° for Ar-TiO_2_ and I-TiO_2_ samples, respectively. The results show that *stable* tetragonal rutile phase (JCPDS 00-004-0551) is the dominant crystal structure of the TiO_2_ nanoparticles. However, the peaks corresponding to anatase phase were not observed [46,47]. By the aid of the Scherer equation [48], crystal sizes were calculated as 54.04 and 58.73 nm for non-modified and plasma-treated catalyst, respectively. 

**Figure 2:**
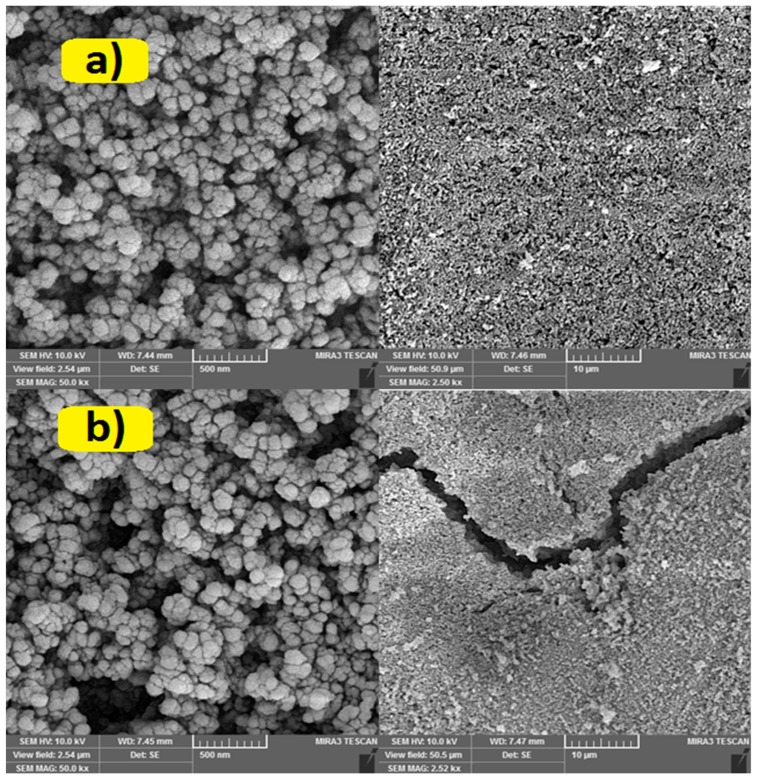
SEM images of I-TiO_2_ (**a**) and Ar-TiO_2_ (**b**) samples at 2.52 kx (left) and 50 kx (right) magnifications.

**Figure 3:**
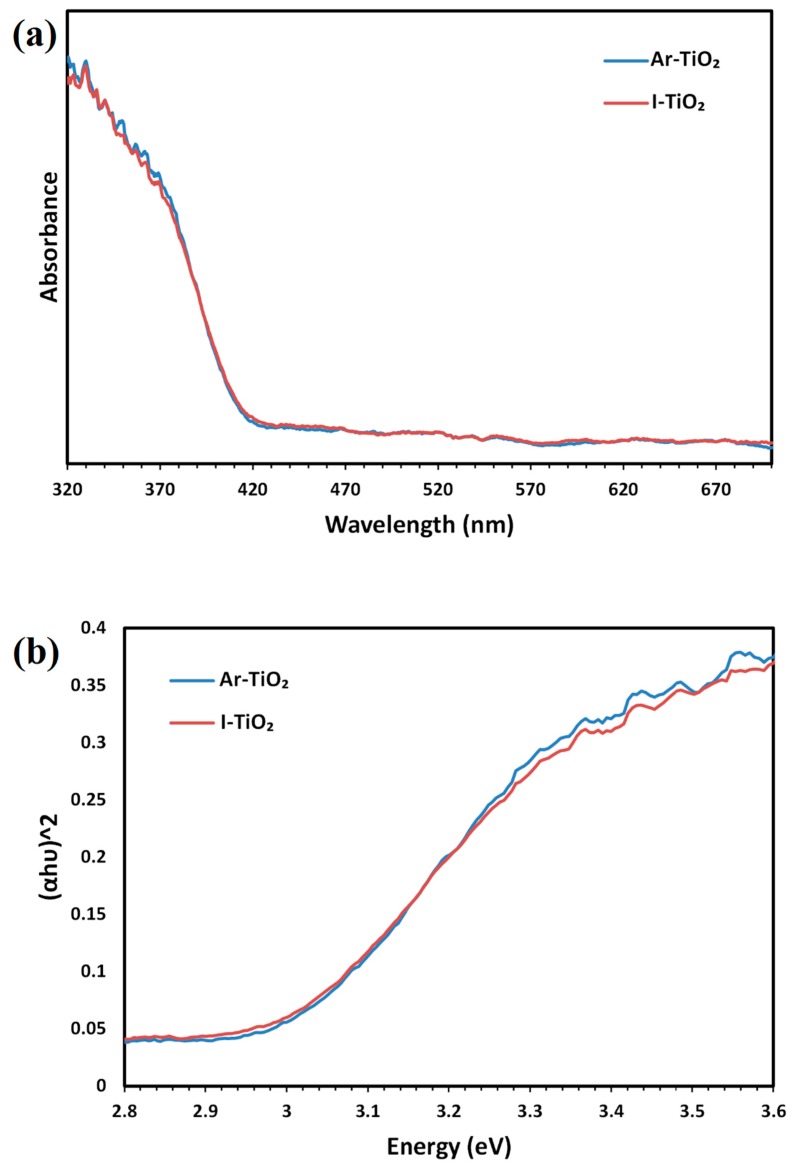
UV-vis DRS of: (**a**) Ar-TiO_2_ and I-TiO_2_ samples and (**b**) plot of (αhv)^2^ as a function of hv for determining the band gap.

Figure 2 shows SEM images of the rutile nano particles immobilized on glass plates before and after Ar plasma treatment. SEM images indicate that the surface of I-TiO_2_ sample is relatively flat (Figure 3a-right), while Ar plasma formed large cracks (Figure 2b-right). Figure 2b-left revealed that entire surface of the treated sample is replete with valleys and cracks (Figure 2a-left). It has been reported that plasma modification was able to generate cracks and cavities on the surface of the catalyst. It is also important to note that plasma only affects upper monolayers of the surface [34,43,44].

Figure 3a shows UV-vis diffuse reflectance spectra of I-TiO_2_ and Ar-TiO_2_ samples. Optical band gap energy of the samples can be obtained by the Tauc equation [49] (Equation (1)):

(1) $$\alpha \mathrm{hv}=B{\left(\mathrm{hv}-E_{g}\right)}^{n}$$

where α is the optical absorption coefficient, hυ (h = Planck’s constant and υ = frequency) is the photon energy, B is constant, $E_{g}$ is the optical band gap energy (eV) and n is ½ for direct band gap semiconductors. In order to obtain the optical band gap energy of the samples, (αhv)^2^ was plotted versus hv (Figure 3b) and E_g_ was calculated. Optical band gap energy for Ar plasma and non-treated samples were found as 2.94 and 2.97 eV, respectively. Although plasma treatment slightly decreased the band gap energy, difference between the band gap energies is not significant for the rutile phase, which already possess a narrow band gap (~3 eV) [33,50].

**Figure 4:**
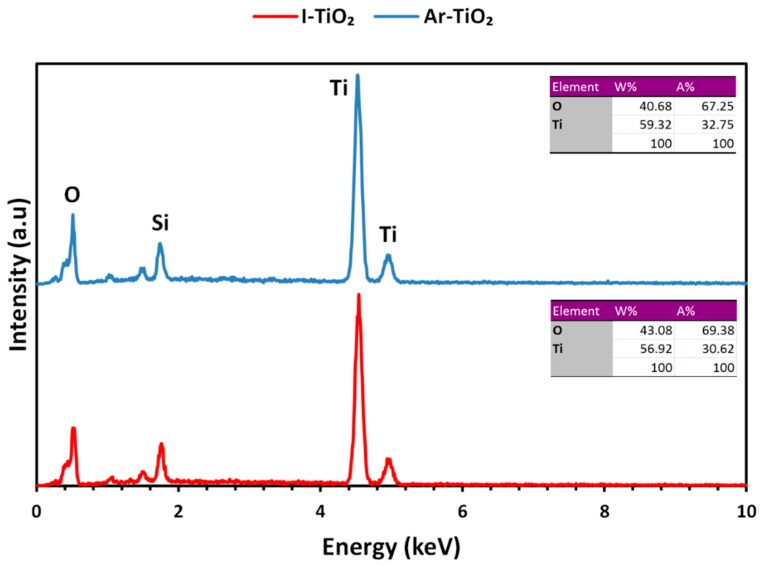
EDX spectrum of plasma-treated and non-treated TiO_2_ nanoparticles immobilized on glass plates.

EDX spectra of the TiO_2_ nanoparticles immobilized on glass plates were collected to investigate the bulk elemental composition of the Ar-TiO_2_ and I-TiO_2_ samples. It can be seen in Figure 4 that plasma treatment slightly reduced atomic percentage of oxygen in the favor of titanium. Also, as it was expected, plasma treatment did not have a significant effect on the bulk structure. It has been reported that cold plasma treatment only modified a few monolayers of the surface, without altering the bulk properties [38,39].

**Figure 5:**
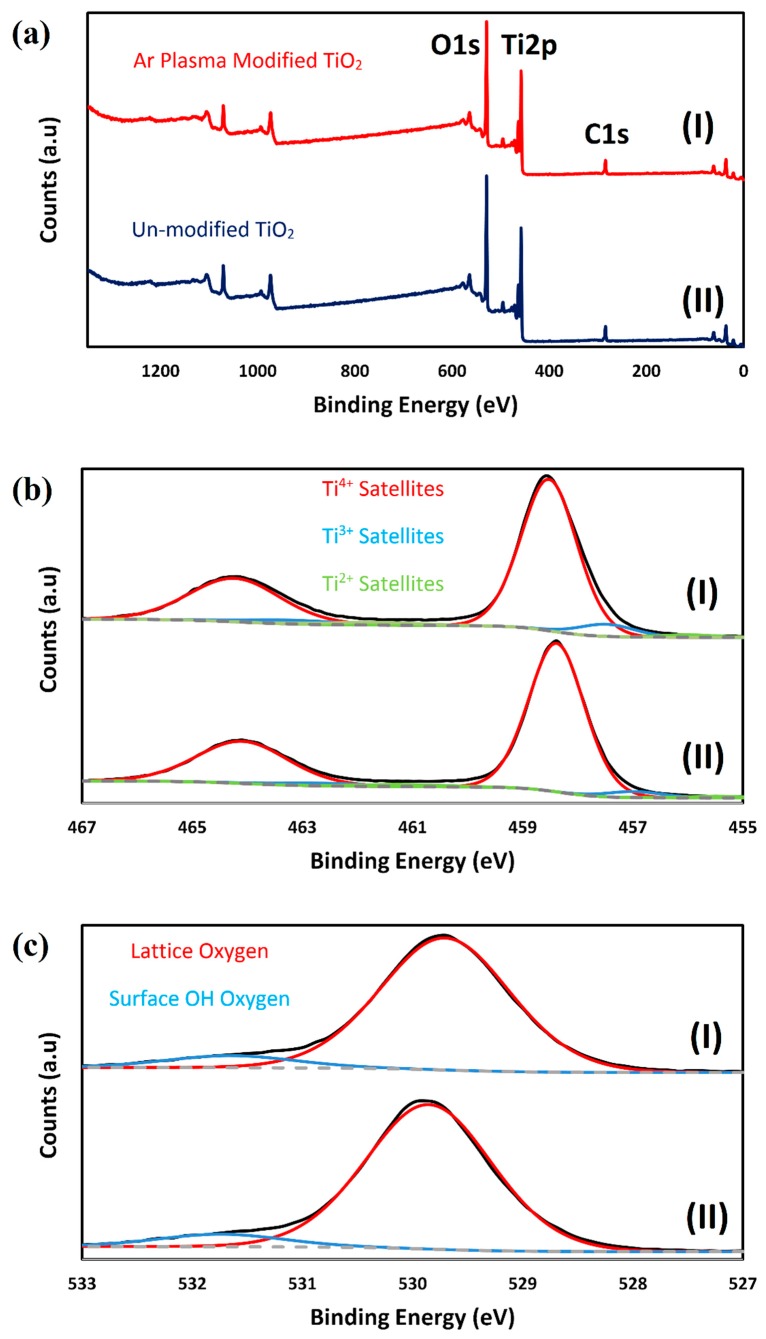
XPS spectra of plasma-treated (I) and non-treated TiO_2_ (II): (**a**) Wide scan spectra; (**b**) HR-XPS spectra of Ti2p; (**c**) HR-XPS spectra of O1s.

XPS analysis was performed to further examine the effect of non-thermal Ar plasma on rutile TiO_2_ nanoparticles. Figure 5 shows XPS spectra of non-immobilized treated and non-treated TiO_2_ nanoparticles. Sharp peaks of C1s, Ti2p and O1s appeared the in the wide-scan spectra for both samples (Figure 5a). High resolution XPS (HR-XPS) spectra of Ti2p are depicted in Figure 5b. The two broad peaks shown in the spectrum of the unmodified sample at 464.09 and 458.38 eV are attributed to Ti2p1/2 and Ti2p3/2, respectively. After Ar plasma treatment, atomic percentages of Ti^3+^ and Ti^2+^ increased by 91.62 and 49.51%, respectively (increasing from 3.82% to 7.32% for Ti^3+^ and from 1.03% to 1.54% for Ti^2+^); while, atomic percentage was reduced by 4.4% for tetravalent Ti^4+^, which is in agreement with the results observed by Bharti et al. [51]. Therefore, oxidation state of Ti decreased after the plasma treatment. HR-XPS spectra of O1s are presented in Figure 5c. Deconvolution of the O1s peak revealed the presence of lattice and non-lattice/surface OH oxygen at 529.71 and 531.65 eV, respectively. Contribution of the non-lattice/surface OH oxygen increased slightly from 7.94 to 8.34% as a result of Ar plasma treatment. 

Ti^3+^ surface defects are some of the most important surface defects in TiO_2_ and an important reactive agent for many adsorbents. Hence, many surface reactions are influenced by these point defects. Sirisuk et al. reported that Ti^3+^ sites play an essential role in photocatalytic process over TiO_2_ photocatalyst [52]. The reported methods to produce TiO_2_ containing Ti^3+^ surface defect include UV irradiation [53,54], heating TiO_2_ under vacuum [55], thermal annealing to high temperatures (above 500 K) [56], and high-energy particle (neutron, Ar^+^, electron, or γ-ray) bombardment [57,58,59]. In this research, Ar plasma was generated Ti^3+^ surface defect by changing Ti^4+^ to Ti^3+^.

### 2.2. Optimal Plasma Treatment Conditions

Decolorization experiments were carried out based on the central composite design and the results are presented in Table 2. The response values (i.e., decolorization percentages) were fitted to the quadratic response function and the coefficients were determined by regression analysis. The obtained regression equation is shown in Equation (2):

(2) $$Y=84.6920-1.9606X_{1}+6.5306X_{2}+0.3046X_{1}^{2}-1.7729X_{2}^{2}-0.4300X_{1}X_{2}$$

where Y is MG decolorization after 30 min of operation using each catalyst configuration, while X_1_ and X_2_ are coded values of plasma pressure and exposure time, respectively. A positive sign for the coefficients in the fitted model indicates that the decolorization efficiency increases with increase in the level of the corresponding factor.

**Figure 6:**
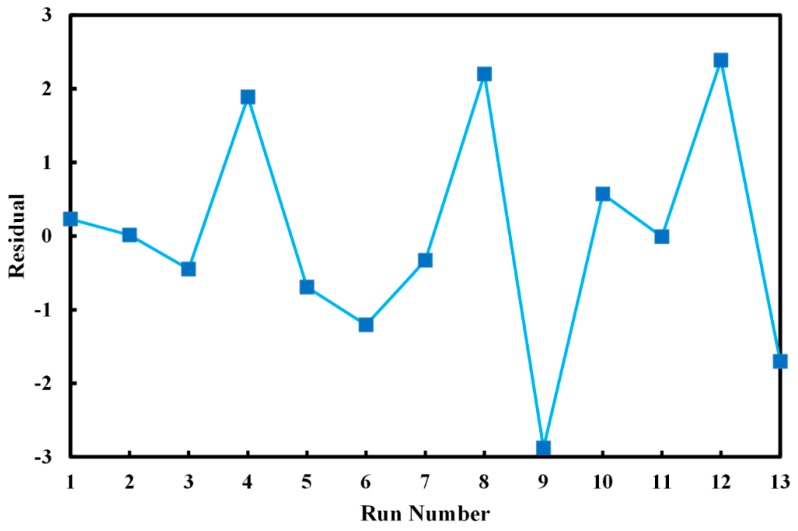
Residuals of photocatalytic MG decolorization after 30 min in each experiment by plasma-treated catalysts.

The statistical significance of the presented quadratic model was examined using analysis of variances (ANOVA). Coefficient of determination (R^2^) was found to be 93.42%, which means that a high level of variation of MG decolorization was explained by the proposed model. Also, the value of adjusted coefficient of determination (R^2^_adj_) was found to be 88.73%, which is close enough to the coefficient of determination and proves the significance of the model. The residuals plot against run number is depicted in Figure 6. According to this figure, residuals are distributed unevenly between experiments, confirming the reliability of obtained model [60]. Results of ANOVA analysis are tabulated in Table 3. The F-value of the model was 19.89, showing that proposed model was highly significant, which is double confirmed by the low P-value of 0.001. In order to study the effects of exposure time and plasma pressure, significance test of quadratic equation constants was performed and presented in Table 4. High F-value of 9.250 and P-value of 0.000 for B_2_ term, which corresponds for plasma pressure, proves that pressure plays the main role in the decolorization efficiency of our plasma-treated photocatalyst.

**Figure 7:**
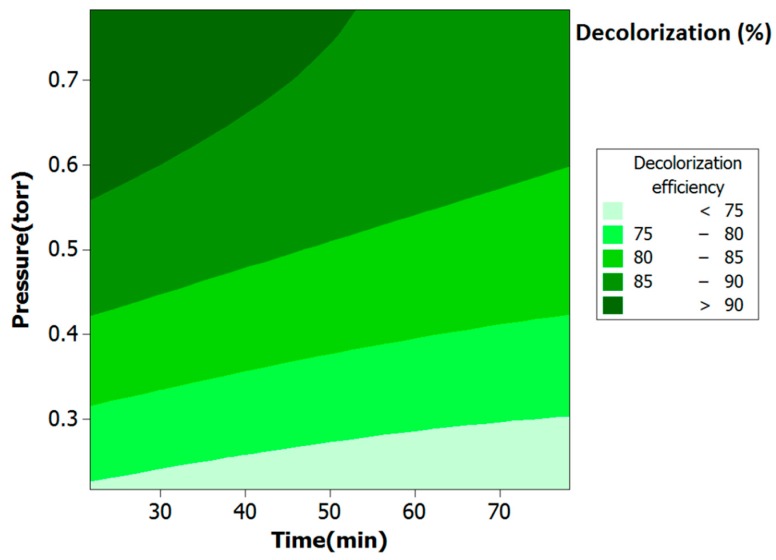
Response surface of MG decolorization as a function of plasma exposure time and pressure.

Figure 7 depicts a visual presentation of the correlation of the decolorization efficiency to plasma exposure time and pressure. Increase of plasma operation pressure led to higher decolorization percentage, while increase of plasma exposure time influenced the decolorization negatively. Operation pressure of the plasma treatment is inversely proportional to the plasma sheath thickness. The plasma sheath thickness is directly proportional to the velocity of Ar particles and the electrical field. Therefore, decrease of the pressure increases the thickness of the plasma sheath, which subsequently enhances the velocity and the electrical field [61]. Increase of the electrical field induces the electrons to collide with the surface of TiO_2_ with more power [62], damaging the surface of the nanoparticles. Therefore, higher pressure reduces the electric field and prevents the surface from being damaged. On the other hand, higher plasma exposure time increases collision of the electrons with the surface. These collisions damage the structure of TiO_2_ and reduce the photocatalytic activity.

Optimal operating conditions of the cold plasma treatment were predicted to be 21.7 min and 0.78 Torr for plasma exposure time and pressure, respectively. MG decolorization efficiency after 30 min was predicted to be 94.62% at these conditions, which is in a good agreement with the obtained experimental value of 94.94%. As expected, the optimized decolorization efficiency is higher than the decolorization percentage at any random conditions. For instance, the photocatalyst treated with Ar plasma for 50 min at a pressure of 0.22 Torr eliminates 69.03% MG solution after 30 min, which is very lower than the optimal decolorization efficiency of 94.94%.

### 2.3. Decolorization Efficiency of Treated Catalyst vs. Non-Treated Catalyst 

**Figure 8:**
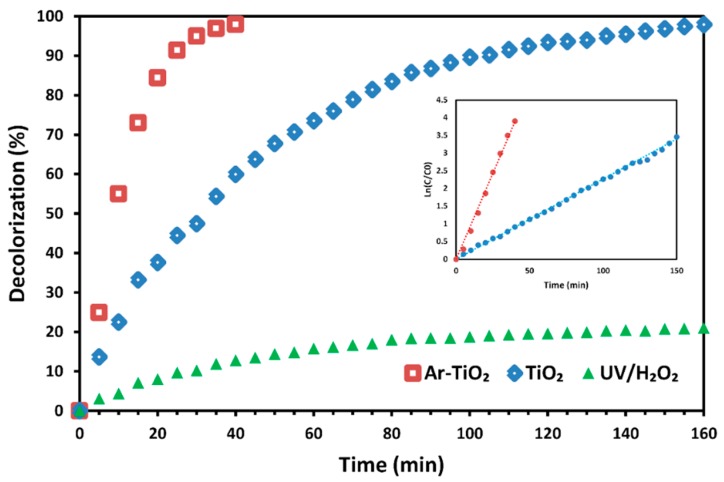
MG decolorization efficiency of plasma-treated and non-treated TiO_2_ nanoparticles immobilized on glass plates; initial reaction conditions: [MG] = 10 mg/L and [H_2_O_2_] = 13 mM.

Figure 8 compares MG decolorization efficiency of the Ar-TiO_2_ sample, treated at the optimum plasma treatment conditions, with that of I-TiO_2_ photocatalyst and UV/H_2_O_2_ process in the absence of titanium dioxide. After 160 min of UVA irradiation in the presence of hydrogen peroxide, the decolorization efficiency reached 21%, which was boosted to 98% by the addition of TiO_2_-immobilized (I-TiO_2_) glass plate. MG elimination using the optimized Ar-TiO_2_ sample reached 98% after 40 min, while I-TiO_2_ needed 160 min to achieve the same decolorization percentage. Kinetics of the MG decolorization can be expressed using the pseudo first order kinetic model [57]:

(3) $$-r_{\mathrm{dye}}=k_{\mathrm{obs}}\times C_{\mathrm{dye}}$$

where r_dye_, k_obs_ and C_dye_ are decolorization rate, rate constant and dye concentration, respectively. Pseudo first order kinetic rate constant was 0.0972 min^−1^ for the optimized Ar-TiO_2_ (R^2^ = 99.28%), which is 4.28 times greater than the rate constant (k_obs_ = 0.0227 min^−1^) of the I-TiO_2_ photocatalyst (R^2^ = 99.64%).

**Figure 9:**
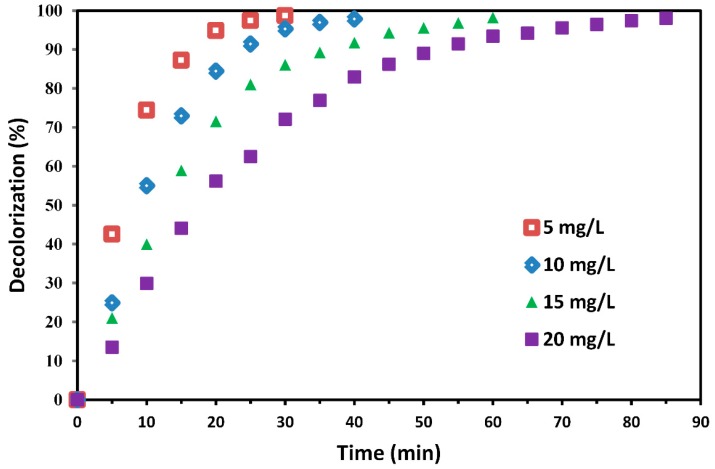
MG decolorization efficiency of TiO_2_-Ar catalyst at various initial dye concentrations; [H_2_O_2_] = 13 mM.

**Figure 10:**
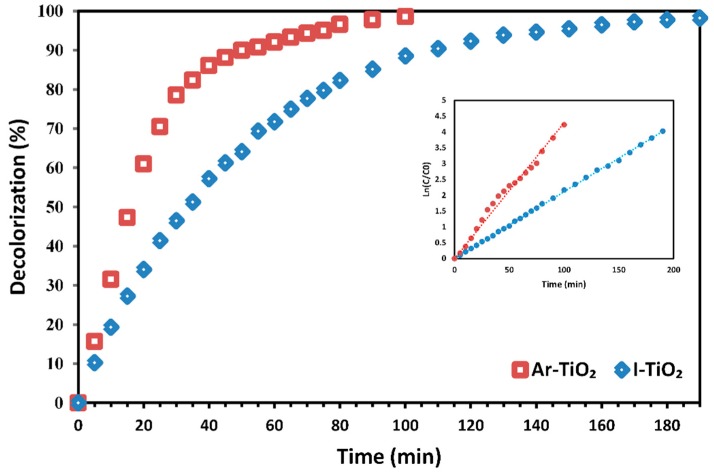
Crystal violet decolorization efficiency of plasma-treated and non-treated TiO_2_ nanoparticles immobilized on glass plates; initial reaction conditions: [CV] = 10 mg/L and [H_2_O_2_] = 13 mM.

In order to study the effect of initial concentration of MG on the decolorization efficiency, additional experiments were performed at various concentrations of MG (Figure 9). Decolorization efficiencies of 5, 10, 15 and 20 mg/L initial dye concentrations were about 98% at different irradiation times of 30, 40, 60 and 85 min, respectively. It is clear from Figure 10 that after 30 min, the decolorization efficiency decreased from 98.7% to 72.1% with increasing dye concentration from 5 to 20 mg/L. Increase in MG concentration, decreases the ratio of available hydroxyl radicals to dye molecules, which results in a decreased degradation percentage. Another possible reason for this behavior is that at higher dye concentrations more organic pollutants are adsorbed on TiO_2_ surface; a large number of dye molecules shield the UV light, while fewer photons reach the TiO_2_ surface and fewer hydroxyl radicals are formed. Consequently, the photocatalytic degradation is decreased [63,64].

To demonstrate the catalytic activity of surface modified photocatalyst in decolorization of other dyestuff, treated and unmodified photocatalysts were used to decolorize the crystal violet (CV). The experiments were performed at fixed initial dye concentration of 10 mg/L and H_2_O_2_ concentration of 13 mM (similar to the MG decolorization conditions). Figure 10 demonstrates that the decomposition of CV in the presence of the optimized Ar-TiO_2_ sample reached 98% within 100 min, while the decolorization with the I-TiO_2_ photocatalyst needed about 190 min to reach the same decolorization percentage. A pseudo first order kinetic rate constant of 0.0429 min^−1^ was obtained for the treated photocatalyst (R^2^ = 98.65%), which is 2.03 times higher than the rate constant (k_obs_ = 0.0211 min^−1^) of the non-treated photocatalyst (R^2^ = 99.95%).

The superior performance of the Ar-TiO_2_ catalyst can be explained using the differences in characteristics of the treated and non-treated catalysts. Interaction of plasma with titanium dioxide is a complicated process due to the presence of ions, photons, radicals and electrons in various excited energy levels. Applying electrical current to the electrodes in the plasma chamber ionizes the argon gas molecules to electrons and positive ions (Equation (4)) [56]. Electrons can subsequently participate in a reaction with titanium dioxide (Equation (5)), generating oxygen vacancy on the surface of the catalyst [58].

(4) $$\mathrm{Ar}\to {\mathrm{Ar}}^{+}+e^{-}$$

(5) $${\mathrm{Ti}}^{4+}:1s^{2}/2s^{2}2p^{6}/3s^{2}3p^{6}/4s^{0}3d^{0}4p^{0}+e^{-}\to {\mathrm{Ti}}^{3+}:1s^{2}/2s^{2}2p^{6}/3s^{2}3p^{6}/4s^{1}3d^{0}4p^{0}$$

The results presented in Section 3.1. showed that Ar plasma treatment did not alter the crystalline phase of rutile TiO_2_ nanoparticles, which is the most thermodynamically stable phase of natural titania. The UV-DRS analyses showed that the band gap energy also remained almost unchanged and only decreased by a small margin. However, XPS analyses demonstrated an increase in Ti^3+^ molar ratio and a decline of oxygen to titanium ratio, which resulted in oxygen and titanium defects on the surface of the catalyst. Surface oxygen vacancies can act as active sites and enhance activity of the titanium dioxide as photocatalyst [30,59]. Furthermore, the observed increase of surface hydroxyl group aids the formation of hydroxyl radicals, increasing the degradation rate of organic pollutants [65].

Pan et al. [59] have found that adsorption of oxygen molecule on perfect neutral titanium dioxide surface is not possible. They have reported that oxygen is adsorbed only on a surface with excess negative charge or when there is oxygen vacancy. Due to the existence of oxygen vacancies, adsorbed O_2_ and H_2_O molecules capture photo-generated electrons and improve charge transportation. This generates highly reactive species such as superoxide radical anions ($O_{2}^{-}$) and hydroxyl radicals [60] which subsequently decolorize the solutions by oxidizing dye molecules.

Another reason for increased performance of the plasma-treated samples may be related to increased surface area of the optimized Ar-TiO_2_ sample. Khataee et al. reported change in the physical surface structure of Ar cold plasma-treated pyrite [61,62]. As it was shown in Figure 3, Ar plasma-treated surface poses cracks over the surface. This phenomenon may result in an increase in effective surface available for photocatalytic reactions, improving its overall performance.

### 2.4. Reusability of Plasma-Modified Catalyst

**Figure 11:**
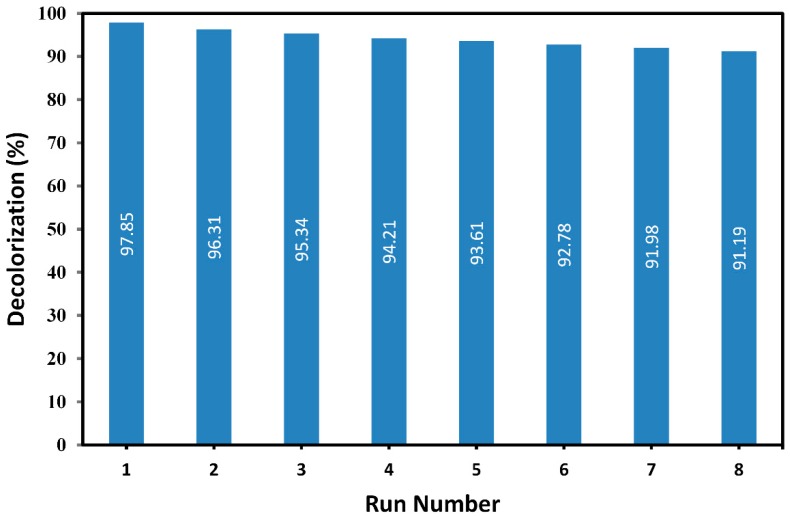
Decolorization efficiency of MG solution in consecutive runs after 40 min; [MG] = 10 mg/L and [H_2_O_2_] = 13 mM.

Another important factor in determination of performance of the catalyst is its ability to maintain its reaction efficiency over time. In order to evaluate the reusability of Ar-TiO_2_ sample, a single glass plate with immobilized plasma-modified rutile TiO_2_ was utilized in UV/TiO_2_/H_2_O_2_ process to decolorize a 10 mg/L MG solution for 8 cycles of 40 min. After each cycle, the Ar-TiO_2_ sample was thoroughly rinsed with deionized water and dried in an oven at a temperature of 70 °C. Figure 11 demonstrates the reusability results after 8 consecutive cycles. 

The results showed that decolorization efficiency dropped from 97.85% after 40 min of operation in the first run to 91.19% after eight consecutive runs. The relative performance of the catalyst was 93.19% after a total of 320 min photocatalytic decolorization process. Therefore, Ar-TiO_2_ photocatalyst was able to maintain a high performance level in a long run and its reusability results were promising.

## 3. Materials and Methods 

### 3.1. Materials

TiO_2_ nanoparticles (rutile phase, 99.9%, 30 nm) were purchased from US Research Nanomaterials Co. (Houston, TX, USA) Malachite green oxalate and crystal violet were obtained from Sigma Aldrich (St. Louis, MO, USA). H_2_O_2_ (30–35%) and NaOH were supplied from Mojallali Co. Ltd. (Tehran, Iran). HCl (37%) was purchased from Merck (Darmstadt, Germany). All solutions were prepared with distilled water.

### 3.2. Apparatus

A 6 W UV-A lamp (F6T5, Hitachi, Tokyo, Japan) provided the irradiation. A UV–vis spectrophotometer (1700 UV–vis Shimadzu, Kyoto, Japan) was used to determine dye concentration. Scanning electron microscopy (SEM) images and EDX analysis were carried out using MIRA3FEG-SEM (TescanBrno, Brno, Czech Republic). X-ray diffraction (XRD) analysis was performed using a D5000 system (Siemens, Berlin, Germany) equipped with a copper anode (λ = 0.15406 nm). UV-vis Diffuse Reflectance Spectra (UV-DRS) were collected using an Avaspec-2048-TEC spectrometer (Avantes, Apeldoorn, Netherlands). X-ray Photoelectron Spectroscopy (XPS) analysis was performed using a K-Alpha spectrometer (Thermo Scientific, Waltham, MA, USA) with an aluminum anode (Al Kα = 1468.3 eV) at an electron take-off angle of 90. The binding energy scale was calibrated by assigning the C1 s peak at 284.5 eV. A Sonoplus ultrasonic homogenizer (Sonic 6D, James Products, Inc., Northampton, England) was applied for sonication. pH value of the solutions was measured with a PHT-110 pH-meter (Labtron, Tehran, Iran). 

### 3.3. Immobilization of TiO_2_ and Plasma Treatment

The heat attachment method was used to immobilize TiO_2_ nanoparticles on glass plates. Surface of glass plates was modified with hydroxyl groups by soaking in NaOH solution (1N) for 2 h. Then, 0.5 g of rutile TiO_2_ nanoparticles was suspended in 50 mL of distilled water. The desired amount of HCl (1N) was injected into the solution to obtain a pH value of 3. After 30 min of sonication, the solution was poured onto glass plates and allowed to dry at 80 °C. Finally, the plates were washed with distilled water and put at 480 °C for 5 h to complete the heat attachment process. This sample is called I-TiO_2_ in this paper.

**Figure 12:**
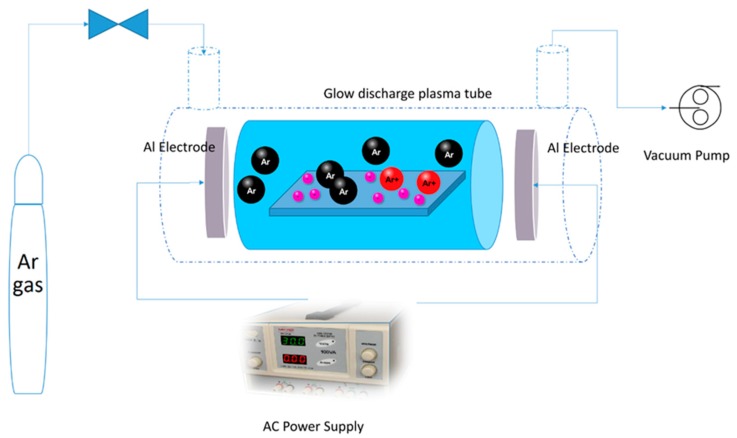
Schematic of non-thermal glow discharge plasma setup.

To modify the surface of rutile TiO_2_ nanoparticles immobilized on glass plates, cold plasma in Argon atmosphere was implemented. Schematic of the plasma treatment setup is shown in Figure 12. The chamber of plasma reactor is made of Pyrex tube sealed with two aluminum bonnets on both sides. Rotary and molecular pumps were used to reach low pressures. The plasma reactor was flushed several times with Argon gas to remove any impurities in the chamber. The flow of gas was adjusted with a mass flow controller and alternative current (AC) was applied to aluminum electrode to generate cold plasma. The plasma-modified sample is referred to as Ar-TiO_2_ in this paper.

### 3.4. Photocatalytic Batch Reactor

Photochemical decolorization was conducted in a rectangular batch reactor (3 cm × 3 cm × 21 cm) containing TiO_2_ nanoparticles immobilized on glass plates. A glass plate (3 cm × 3 cm × 20 cm) containing 0.16 g of immobilized rutile TiO_2_ nanoparticles was placed at the bottom of the reactor for each experiment. The UV lamp was placed at the top of the reactor to provide irradiation. The batch reactor was filled with 100 mL of dye solution (10 mg/L) and H_2_O_2_ (13 mM) was injected into the dye solution at the beginning of each experiment. This specific concentration of H_2_O_2_ was determined by performing preliminary MG decolorization experiments to maximize the efficiency of the process at a MG concentration of 10 mg/L and various H_2_O_2_ concentrations from 5 to 15 mM was examined. Decolorization process was carried out in a shaker incubator at 25 °C and a rotation speed of 10 rpm. Dye concentration was monitored by a UV spectrophotometer at the maximum wavelength of 618 nm and 595 nm for Malachite green and Crystal violet, respectively. Decolorization percentage was calculated using the following equation (Equation (6)) [66]:

(6) $$\mathrm{Decolorization}\;(\%)=(1-\frac{C_{t}}{C_{0}})\times 100$$

where C_0_ and C_t_ are the concentrations of the sample solution at times 0 and t, respectively.

### 3.5. Response Surface Methodology and Experimental Design

Experimental design and optimization were carried out using response surface methodology by the aid of Minitab 16.2 software (Minitab Inc., State College, PA, USA). Each independent variable was coded as ‘X_i_’ using the following equation (Equation (7)):

(7) $$X_{i}=\frac{x_{i}-x_{0}}{\delta x}$$

where X_i_ is the coded value, x_0_ denotes the value of x_i_ at the center point and δx is the step change value.

The plasma exposure time (X_1_) and pressure (X_2_) are the critical factors in preparing the plasma-treated photocatalyst. Optimization of these factors will increase the efficiency of the photocatalyst and maximize the decolorization. The decolorization percentage after 30 min of reaction in presence of H_2_O_2_ (13 mM) was chosen as the response (i.e., dependent variable). Each codified variable was studied at five levels (−1.41, −1, 0, 1, +1.41). Table 5 shows levels and values of coded variables. Ar glow discharge plasma formed in pressures below 0.8 Torr (1 Torr = 133.32 Pa), therefore the upper limit for plasma pressure was set to be 0.78 Torr in our design. A second order polynomial Equation (8) was employed to correlate the response to the independent variables X_1_ and X_2_:

(8) $${Y=B}_{0}+\sum_{i=1}^{n}B_{i}X_{i}+\sum_{i=1}^{n}B_{\mathrm{ii}}X_{i}^{2}+\sum_{\begin{array}{l} i,j=1\\ j\neq i \end{array}}^{n}B_{\mathrm{ij}}X_{\mathrm{ij}}$$

where Y is the predicted response, B_0_ is the interception term, B_i_ are the linear coefficients, B_ii_ are the quadratic coefficients, B_ij_ are the interaction coefficients, X_i_ are the coded variables and n is the number of independent variables.

## 4. Conclusions

In this study, the cold plasma treatment was applied to modify the surface properties of immobilized rutile TiO_2_ nano particles on the glass plates. A five-level central composite design was employed to model and optimize the photocatalytic decolorization efficiency by considering the plasma exposure time and pressure as the independent variables, and the decolorization efficiency as the dependent variable using response surface methodology. Results showed that optimizing plasma pressure and exposure time (parameters that are usually overlooked) had significant effect on enhancing activity of the photocatalyst. Plasma pressure was found to be the most important parameter to adjust, and its increase, enhanced decolorization efficiency. On the other hand, increase of plasma exposure time decreased the decolorization efficiency. The optimum values of the exposure time and pressure of plasma treatment process were 21.7 min and 0.78 Torr, respectively. The plasma treatment decreased the oxygen to titanium ratio and created oxygen vacancy on the surface of the catalyst. As a result, the plasma-treated catalyst showed superior performance than the non-treated catalyst in decolorization of the aqueous solutions of Malachite green and Crystal violet. It can be concluded that cold plasma is an effective method for enhancing the photocatalytic performance of rutile titanium dioxide without addition of any impurities. The Ar plasma-modified rutile phase maintained its low band gap energy, making it a better competitor for anatase titanium dioxide in visible light operations.

## Figures and Tables

**Table 1:** Molecular structures of the malachite green and crystal violet dyes.

Dye Name	Chemical Structure	Molecular Formula	Color Index Number	λ_max_ (nm)	Mw
Malachite Green Oxalate	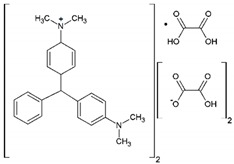	C_52_H_54_N_4_O_12_	42000	618	921
Crystal Violet	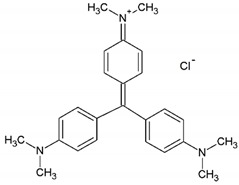	C_25_H_30_N_3_Cl	42555	595	408

**Table 2:** Experimental and predicted response values for the central composite design. The values in brackets are codified variables.

Run No.	Variables (Level)		Decolorization (%)		Residual
	Plasma Exposure Time (min)	Plasma Operation Pressure (Torr)	Experimental	Predicted	
**1**	50.00 (0)	0.50 (0)	84.92	84.69	0.23
**2**	78.28 (+1.41)	0.50 (0)	82.54	82.53	0.01
**3**	50.00 (0)	0.50 (0)	84.24	84.69	−0.45
**4**	70.00 (+1)	0.30 (−1)	77.05	75.16	1.89
**5**	21.71 (−1.41)	0.50 (0)	87.38	88.07	−0.69
**6**	30.00 (−1)	0.70 (+1)	90.94	92.14	−1.20
**7**	50.00 (0)	0.50 (0)	84.36	84.69	−0.33
**8**	50.00 (0)	0.78 (+1.41)	92.58	90.38	2.10
**9**	50.00 (0)	0.22 (−1.41)	69.03	71.91	−2.88
**10**	50.00 (0)	0.50 (0)	85.26	84.69	0.57
**11**	50.00 (0)	0.50 (0)	84.68	84.69	−0.01
**12**	30.00 (−1)	0.30 (−1)	80.61	78.22	2.39
**13**	70.00 (+1)	0.70 (+1)	85.66	87.36	−1.70

**Table 3:** Analysis of variances for photocatalytic MG decolorization using plasma-treated immobilized TiO_2_ photocatalyst.

Source of Variation	Sum of Squares	Degree of Freedom	Mean Square	*F*-Value	*P*-Value
Model	396.57	5	79.31	19.89	0.001
Residuals	27.91	7	3.98	-	-
Total	424.48	12	-	-	-

**Table 4:** Estimated quadratic equations coefficients for photocatalytic MG decolorization using plasma-treated immobilized TiO_2_ photocatalyst and their respective *F* and *P* values.

Quadratic Term	Estimated Coefficient	Standard Error	*F*-Value	*P*-Value
B_0_ (constant)	84.692	0.893	94.838	0.000
B_1_ (time)	−1.960	0.706	−2.777	0.027
B_2_ (pressure)	6.530	0.706	9.250	0.000
B_11_ (time × time)	0.304	0.757	0.402	0.699
B_22_ (pressure × pressure)	−1.772	0.757	−2.342	0.052
B_12_ (time × pressure)	−0.430	0.998	−0.431	0.680

**Table 5:** Range and level of plasma treatment parameters in CCD design.

Code	Parameter	Level				
		−1.41	−1	0	1	1.41
X_1_	Time (min)	21.71	30	50	70	78.28
X_2_	Pressure (Torr)	0.21	0.3	0.5	0.7	0.78

## References

[1] Baghdadi M., Alipour Soltani B., Nourani M. (2017). Malachite green removal from aqueous solutions using fibrous cellulose sulfate prepared from medical cotton waste: Comprehensive batch and column studies. J. Ind. Eng. Chem..

[2] Zolfaghari P., Shojaat R., Karimi A., Saadatjoo N. (2018). Application of fluidized bed reactor containing GOx/MnFe_2_O_4_/calcium alginate nano-composite in degradation of a model pollutant. J. Environ. Chem. Eng..

[3] Zolfaghari P., Aghbolaghy M., Karimi A., Khataee A. (2019). Continuous degradation of an organic pollutant using heterogeneous magnetic biocatalyst and CFD analysis of the process. Process Saf. Environ. Prot..

[4] Elhami V., Karimi A., Aghbolaghy M. (2015). Preparation of heterogeneous bio-Fenton catalyst for decolorization of Malachite Green. J. Taiwan Inst. Chem. Eng..

[5] Arimi M.M. (2017). Integration of Fenton with biological and physical–chemical methods in the treatment of complex effluents: A review. Environ. Technol. Rev..

[6] Khataee A.R., Fathinia M., Aber S. (2011). Kinetic study of photocatalytic decolorization of C.I. Basic Blue 3 solution on immobilized titanium dioxide nanoparticles. Chem. Eng. Res. Des..

[7] Montalvo-Romero C., Aguilar-Ucán C., Alcocer-Dela hoz R., Ramirez-Elias M., Cordova-Quiroz V. (2018). A Semi-Pilot Photocatalytic Rotating Reactor (RFR) with Supported TiO_2_/Ag Catalysts for Water Treatment. Molecules.

[8] Yaparatne S., Tripp C.P., Amirbahman A. (2018). Photodegradation of taste and odor compounds in water in the presence of immobilized TiO_2_-SiO_2_ photocatalysts. J. Hazard. Mater..

[9] Karimi A., Aghbolaghy M., Khataee A., Shoa Bargh S. (2012). Use of Enzymatic Bio-Fenton as a New Approach in Decolorization of Malachite Green. Sci. World J..

[10] Bendjabeur S., Zouaghi R., Kaabeche O., Sehili T. (2017). Parameters Affecting Adsorption and Photocatalytic Degradation Behavior of Gentian Violet under UV Irradiation with Several Kinds of TiO_2_ as a Photocatalyst. Int. J. Chem. React. Eng..

[11] Rott E., Kuch B., Lange C., Richter P., Kugele A., Minke R. (2018). Removal of Emerging Contaminants and Estrogenic Activity from Wastewater Treatment Plant Effluent with UV/Chlorine and UV/H_2_O_2_ Advanced Oxidation Treatment at Pilot Scale. Int. J. Environ. Res. Public Health.

[12] Shuang S., Zhang Z. (2018). The Effect of Annealing Treatment and Atom Layer Deposition to Au/Pt Nanoparticles-Decorated TiO_2_ Nanorods as Photocatalysts. Molecules.

[13] Kim H.-i., Kim J., Kim W., Choi W. (2011). Enhanced Photocatalytic and Photoelectrochemical Activity in the Ternary Hybrid of CdS/TiO_2_/WO_3_ through the Cascadal Electron Transfer. J. Phys. Chem. C.

[14] Ratanatawanate C., Tao Y., Balkus K.J. (2009). Photocatalytic Activity of PbS Quantum Dot/TiO_2_ Nanotube Composites. J. Phys. Chem. C.

[15] Lee S.-Y., Park S.-J. (2013). TiO_2_ photocatalyst for water treatment applications. J. Ind. Eng. Chem..

[16] Sayılkan F., Asiltürk M., Tatar P., Kiraz N., Şener S., Arpac E., Sayılkan H. (2008). Photocatalytic performance of Sn-doped TiO_2_ nanostructured thin films for photocatalytic degradation of malachite green dye under UV and VIS-lights. Mater. Res. Bull..

[17] Asiltürka M., Sayılkana F., Arpac E. (2009). Effect of Fe^3+^ ion doping to TiO_2_ on the photocatalytic degradation of Malachite Green dye under UV and vis-irradiation. J. Photochem. Photobiol. A Chem..

[18] Rajabi H.R., Khani O., Shamsipur M., Vatanpour V. (2013). High-performance pure and Fe^3+^-ion doped ZnS quantum dots as green nanophotocatalysts for the removal of malachite green under UV-light irradiation. J. Hazard. Mater..

[19] Kaneva N., Stambolova I., Blaskov V., Dimitriev Y., Vassilev S., Dushkin C. (2010). Photocatalytic activity of nanostructured ZnO films prepared by two different methods for the photoinitiated decolorization of malachite green. J. Alloys Compd..

[20] Ameen S., Shaheer Akhtar M., Nazim M., Shin H.-S. (2013). Rapid photocatalytic degradation of crystal violet dye over ZnO flower nanomaterials. Mater. Lett..

[21] Chen C.-C., Mai F.-D., Chen K.-T., Wu C.-W., Lu C.-S. (2007). Photocatalyzed *N*-de-methylation and degradation of crystal violet in titania dispersions under UV irradiation. Dyes Pigments.

[22] Li W., Bak T., Atanacio A., Nowotny J. (2016). Photocatalytic properties of TiO_2_: Effect of niobium and oxygen activity on partial water oxidation. Appl. Catal. B.

[23] Jung C.-K., Bae I.-S., Song Y.-H., Boo J.-H. (2005). Plasma surface modification of TiO_2_ photocatalysts for improvement of catalytic efficiency. Surf. Coat. Technol..

[24] An H.-R., Park S.Y., Kim H., Lee C.Y., Choi S., Lee S.C., Seo S., Park E.C., Oh Y.-K., Song C.-G. (2016). Advanced nanoporous TiO_2_ photocatalysts by hydrogen plasma for efficient solar-light photocatalytic application. Sci. Rep..

[25] Khataee A., Rad T.S., Vahid B., Khorram S. (2016). Preparation of zeolite nanorods by corona discharge plasma for degradation of phenazopyridine by heterogeneous sono-Fenton-like process. Ultrason. Sonochem..

[26] Dastan D. (2017). Effect of preparation methods on the properties of titania nanoparticles: Solvothermal versus sol-gel. Appl. Phys. A.

[27] Dastan D., Chaure N., Kartha M. (2017). Surfactants assisted solvothermal derived titania nanoparticles: Synthesis and simulation. J. Mater. Sci. Mater. Electron..

[28] Attri P., Arora B., Choi E.H. (2013). Retracted Article: Utility of plasma: A new road from physics to chemistry. RSC Adv..

[29] Yavuz Ö., Saka C. (2013). Surface modification with cold plasma application on kaolin and its effects on the adsorption of methylene blue. Appl. Clay Sci..

[30] Zhou R., Zhou R., Zhang X., Li J., Wang X., Chen Q., Yang S., Chen Z., Bazaka K., Ostrikov K.K. (2016). Synergistic Effect of Atmospheric pressure Plasma and TiO_2_ Photocatalysis on Inactivation of Escherichia coliCells in Aqueous Media. Sci. Rep..

[31] Dastan D., Londhe P.U., Chaure N.B. (2014). characterization of TiO_2_ nanoparticles prepared using different surfactants by sol-gel method. J. Mater. Sci: Mater. Electron..

[32] Manolache S., Romanescu C., Cobileac G., Simionescu C. (1997). Some Terpenes and Related Derivatives Synthesized in Cold Plasma Systems. Molecules.

[33] Castro Vidaurre E.F., Achete C.A., Gallo F., Garcia D., Simão R., Habert A.C. (2002). Surface Modification of Polymeric Materials by Plasma Treatment. Mater. Res..

[34] Quitzau M., Wolter M., Kersten H. (2009). Plasma Treatment of Polyethylene Powder Particles in a Hollow Cathode Glow Discharge. Plasma Process. Polym..

[35] Popelka A., Novák I., Lehocký M., Chodák I., Sedliačik J., Gajtanska M., Sedliačiková M., Vesel A., Junkar I., Kleinová A. (2012). Anti-bacterial Treatment of Polyethylene by Cold Plasma for Medical Purposes. Molecules.

[36] Ma Y., Manolache S., Denes F., Cho J., Timmons R. (2006). Surface Modification of Rutile TiO_2_ by Submerged Arc Discharge for mproved Photoreactivity. J. Mater. Eng. Perform..

[37] Yang Y., Zhang T., Le L., Ruan X., Fang P., Pan C., Xiong R., Shi J., Wei J. (2014). Quick and Facile Preparation of Visible light-Driven TiO_2_ Photocatalyst with High Absorption and Photocatalytic Activity. Sci. Rep..

[38] Shoaebargh S., Karimi A. (2014). RSM modeling and optimization of glucose oxidase immobilization on TiO_2_/polyurethane: Feasibility study of AO7 decolorization. J. Environ. Chem. Eng..

[39] Zeng Z., Zou H., Li X., Sun B., Chen J., Shao L. (2012). Ozonation of acidic phenol wastewater with O_3_/Fe(II) in a rotating packed bed reactor: Optimization by response surface methodology. Chem. Eng. Process. Process Intensif..

[40] Ozbay N., Yargic A.S. (2015). Factorial experimental design for Remazol Yellow dye sorption using apple pulp/apple pulp carbon–titanium dioxide co-sorbent. J. Clean. Prod..

[41] Devi L.G., Nithya P.M., Abraham C., Kavitha R. (2017). Influence of surface metallic silver deposit and surface fluorination on the photocatalytic activity of rutile TiO_2_ for the degradation of crystal violet a cationic dye under UV light irradiation. Mater. Today Commun..

[42] Tao T., Chen Y., Zhou D., Zhang H., Liu S., Amal R., Sharma N., Glushenkov A.M. (2013). Expanding the applications of the ilmenite mineral to the preparation of nanostructures: TiO_2_ nanorods and their photocatalytic properties in the degradation of oxalic acid. Chemistry.

[43] Tang S., Choi H.S. (2008). Comparison of Low- and Atmospheric-Pressure Radio Frequency Plasma Treatments on the Surface Modification of Poly(methyl methacrylate) Plates. J. Phys. Chem. C.

[44] Şahin Ö., Kaya M., Saka C. (2015). Plasma-surface modification on bentonite clay to improve the performance of adsorption of methylene blue. Appl. Clay Sci..

[45] Bharti B., Kumar S., Lee H.-N., Kumar R. (2016). Formation of oxygen vacancies and Ti^3+^ state in TiO_2_ thin film and enhanced optical properties by air plasma treatment. Sci. Rep..

[46] Berger T., Sterrer M., Diwald O., Knözinger E., Panayotov D., Thompson T.L., Yates J.T. (2005). Light-Induced Charge Separation in Anatase TiO_2_ Particles. J. Phys. Chem. B.

[47] Bityurin N., Kuznetsov A.I., Kanaev A. (2005). Kinetics of UV-induced darkening of titanium-oxide gel. Appl. Surf. Sci..

[48] Dastan D., Panahi S.L., Chaure N.B. (2016). Characterization of titania thin films grown by dip coating technique. J. Mater. Sci. Mater. Electron..

[49] Dastan D. (2015). Nanostructured Anatase Titania Thin Films Prepared by Sol-Gel Dip Coating Technique. J. Atom. Mol. Condens. Nano Phys. (JAMCNP).

[50] Dohshi S., Anpo M., Okuda S., Kojima T. (2005). Effect of γ-ray Irradiation on the Wettability of TiO_2_ Single Crystals. Top. Catal..

[51] Henrich V.E., Kurtz R.L. (1981). Surface electronic structure of TiO_2_: Atomic geometry, ligand coordination, and the effect of adsorbed hydrogen. Phys. Rev. B.

[52] Jun J., Dhayal M., Shin J.-H., Kim J.-C., Getoff N. (2006). Surface properties and photoactivity of TiO_2_ treated with electron beam. Radiat. Phys. Chem..

[53] Lu G., Linsebigler A., Yates J.T. (1994). Ti^3+^ Defect Sites on TiO_2_(110): Production and Chemical Detection of Active Sites. J. Phys. Chem..

[54] Hassani A., Soltani R.D.C., Kıranşan M., Karaca S., Karaca C., Khataee A. (2016). Ultrasound-assisted adsorption of textile dyes using modified nanoclay: Central composite design optimization. Korean J. Chem. Eng..

[55] Fridman A. (2008). Plasma Chemistry.

[56] Liberman M.A., Lichtenberg A.J. (2005). Principles of Plasma Discharges and Materials Processing.

[57] Jia Y., Liu J., Cha S., Choi S., Park Y.C., Liu C. (2017). Magnetically separable Au-TiO_2_/nanocube ZnFe_2_O_4_ composite for chlortetracycline removal in wastewater under visible light. J. Ind. Eng. Chem..

[58] Sakata K. (1983). Study of electronic structure of reduced TiO_2_ and VxTi_1_−xO_2_ crystals by ESCA and optical absorption. Phys. Status Solidi B.

[59] Pan X., Yang M.-Q., Fu X., Zhang N., Xu Y.-J. (2013). Defective TiO_2_ with oxygen vacancies: Synthesis, properties and photocatalytic applications. Nanoscale.

[60] Zangeneh H., Zinatizadeh A.A.L., Habibi M., Akia M., Hasnain Isa M. (2015). Photocatalytic oxidation of organic dyes and pollutants in wastewater using different modified titanium dioxides: A comparative review. J. Ind. Eng. Chem..

[61] Khataee A., Bozorg S., Khorram S., Fathinia M., Hanifehpour Y., Joo S.W. (2013). Conversion of Natural Clinoptilolite Microparticles to Nanorods by Glow Discharge Plasma: A Novel Fe-Impregnated Nanocatalyst for the Heterogeneous Fenton Process. Ind. Eng. Chem. Res..

[62] Khataee A., Gholami P., Vahid B. (2016). Heterogeneous sono-Fenton-like process using nanostructured pyrite prepared by Ar glow discharge plasma for treatment of a textile dye. Ultrason. Sonochem..

[63] Yahia Cherif L., Yahiaoui I., Aissani-Benissad F., Madi K., Benmehdi N., Fourcade F., Amrane A. (2014). Heat Attachment Method for the Immobilization of TiO_2_ on Glass Plates: Application to Photodegradation of Basic Yellow Dye and Optimization of Operating Parameters, Using Response Surface Methodology. Ind. Eng. Chem. Res..

[64] Liu C.-C., Hsieh Y.-H., Lai P.-F., Li C.-H., Kao C.-L. (2006). Photodegradation treatment of azo dye wastewater by UV/TiO_2_ process. Dyes Pigments.

[65] Minero C., Mariella G., Maurino V., Pelizzetti E. (2000). Photocatalytic Transformation of Organic Compounds in the Presence of Inorganic Anions. 1. Hydroxyl-Mediated and Direct Electron-Transfer Reactions of Phenol on a Titanium Dioxide-Fluoride System. Langmuir.

[66] Sirisuk A., Klansorn E., Praserthdam P. (2008). Effects of reaction medium and crystallite size on Ti^3+^ surface defects in titanium dioxide nanoparticles prepared by solvothermal method. Catal. Commun..

